# Fracture resistance in endodontically treated tooth using rotary and hand files

**DOI:** 10.6026/973206300210285

**Published:** 2025-03-31

**Authors:** Bisma Jahangeer, Aishwarya Arya, Taniya Elsa Oommen, Kanduri Venkata Naga Vamseekrishna, Divya Mishra, Ena Sharma

**Affiliations:** 1 Department of Conservative Dentistry and Endodontics, MDS - MM College of Dental Sciences & Research, Mullana, Ambala, Haryana, India; 2 Department of Conservative Dentistry and Endodontics, Awadh Dental College and Hospital, Jamshedpur, Jharkhand, India; 4 Department of Conservative Dentistry and Endodontics, Care Dental College, MMCDSR, Mullana, Ambala, Haryana, India; 5 Department of Periodontics, Rayat Bahra Dental College and Hospital, Mohali, Punjab, India

**Keywords:** Fracture resistance, endodontically treated teeth, hand k-files, WaveOne gold, pro-taper next, trunatomy, instrument design, rotary systems, taper, structural integrity

## Abstract

Evaluation and comparison of fracture resistance to endodontically treated teeth prepared with four different file systems (Hand K-Files, Wave One Gold, Pro-Taper Next and TruNatomy) is of interest to dentists. Hence, a total of 50 extracted single-rooted premolars were divided into five groups. The control group and the four groups were then used for biomechanical preparation. The samples were mounted and subjected to a fracture resistance test using a Universal Testing Machine after instrumentation and obturation. Results revealed that the control group exhibited the highest fracture resistance, followed by the Hand K-Files group, TruNatomy, Pro-Taper Next and WaveOne Gold, with the latter showing the lowest resistance. Statistical analysis indicated a significant difference in fracture resistance among the groups (p < 0.0001), highlighting that hand files produced higher fracture resistance compared to the rotary systems. Thus, data shows that instrument design and taper significantly influence the structural integrity of endodontically treated teeth.

## Background:

Endodontic therapy involves the treatment of the endodontic disease by removal of the microorganisms and the infectious agents from the root canal [1]. The long-term success of an endodontic therapy is immensely influenced by how well the coronal and apical seal has been achieved [1]. Biomechanical preparation which involves the elimination of infected, inflamed or necrotic canal tissues in order to create smooth walls to facilitate irrigation and finally the obturation has proved to be the most important step for a successful endodontic therapy [2]. However, tooth fracture due to formation of micro-cracks remains the major complication in such endodontically treated teeth and many studies have found endodontic treatment as the main culprit for tooth fracture 3]. Endodontically treated teeth are more susceptible to fracture than teeth with vital pulp which is primarily attributed to the structural disintegration caused by dental caries and tooth preparation [4]. There is a significant weakening of the tooth structure due to enhanced coronal enlargement obtained with instruments having increased taper which may also lead to fracture of such teeth [5]. Endodontic therapy leads to dentinal dehydration which further makes the teeth brittle [6]. This is predominantly as a result of the changes in the collagen cross-linking of the endodontically treated teeth due to drying over time [7]. Fracture associated with the endodontically treated teeth is one of the most difficult clinical complications that may be affected by a number of factors during of therapy, for instance access cavity preparation, instrumentation with hand or rotary files, undesirable effects of irrigating solutions, excessive amount of pressure applied during the filling and sealing procedures [7] dentin dehydration after the therapy [8] as well as due to variation in the kinematics, design of the instrument and mechanical behaviour [9]. Therefore, it is of interest to report the comparison of fracture resistance of endodontically treated teeth, shaped with nickel-titanium hand K-file (Mani, Japan), Pro-Taper Next (Dentsply, Maillefer, Switzerland), WaveOne Gold (Dentsply, Maillefer, Switzerland) and TruNatomy (Dentsply, Maillefer, Switzerland) after obturation.

## Materials and Methodology:

## Collection and preparation of samples:

50 extracted permanent single rooted premolars were collected from the Department of Oral Surgery, Maharishi Markandeshwar College of Dental Sciences and Research, Mullana, Ambala (Haryana). The samples after extraction were first disinfected with 0.1% Thymol solution and then stored in distilled water at 37°C. The samples were sectioned at/below the cementoenamel junction using a diamond coated disc with a mandrel under water coolant and a uniform length of 16mm was left for each tooth and the teeth were examined under a stereomicroscope under 10X magnification to look for any defects. Teeth with defects were excluded and a total of 50 teeth were selected for the study.

## Instrumentation and obturation:

The total sample size for the study comprised 50 teeth, which were divided into five groups, each consisting of 10 samples. The first group served as the control, while the other four groups were classified based on the file system used for instrumentation. Access to the canals was initiated using an Endo-access diamond bur and an Endo Z carbide bur attached to a high-speed Airotor hand piece with water coolant spray. A straight-line access was prepared to facilitate both instrumentation and obturation. The canals were initially explored with a DG-16 explorer and patency was confirmed using an ISO size 08 K-File. Canal length was measured and apical patency was maintained with an ISO size 10 K-File. The working length was determined to be 0.5 mm short of the apical foramen. All samples, except for the control, were then prepared with hand files until an ISO size 15 K-Flex file bound at the working length.

## The samples were categorized into the following groups based on the technique used:

Group 1 served as the control, with no instrumentation or filling applied to these samples. In Group 2, instrumentation was carried out using the step-back technique with hand K-Files (apical preparation size 35). The canal preparation involved using No. 15 K-Files (0.02 taper), followed by sizes 20, 25, 30, 35 and progressively up to size 50. The master apical file (MAF) size 35 was used in a watch-winding and filing motion. After reaching the working length, step-back filing in 1 mm increments was performed to prepare the middle and coronal thirds of the canal. Recapitulation with the MAF was done at each step and the samples were irrigated with 5.25% sodium hypochlorite after each file, followed by a final rinse with 17% EDTA. In Group 3, the WaveOne Gold system was employed using the crown-down technique. Ten samples were instrumented using the WaveOne Gold reciprocating single-file system with an X-Smart plus Endomotor at a speed of 300 rpm and a torque of 2.5 N/cm^2^. After flaring the canal orifices, a glide path was created using the WaveOne Gold Glider. The canals were prepared with the WaveOne Gold Primary single file (25/0.07 taper) using a brushing motion and 3 mm pecking movements until the working length was achieved. The irrigation protocol was the same as for Group 2. In Group 4, the Pro-Taper Next file system was employed, again using the crown-down technique. Ten samples were instrumented with Pro-Taper Next files using the X-Smart plus Endomotor at 300 rpm and a torque of 2 N/cm^2^. Canals were initially explored with small-sized hand files to establish a reproducible glide path. Subsequently, Pro-Taper Next files (X1 [17/0.04] and X2 [25/0.06]) were used in a gentle in-and-out brushing motion to reach the working length. The irrigation protocol mirrored that of Group 3.

In Group 5, the TruNatomy file system was used, also with the crown-down technique. Ten samples were instrumented using TruNatomy files with the X-Smart plus Endomotor, rotating at 500 rpm with a torque of 1.5 N/cm^2^. The working length was determined using ISO #10 K-File and apical patency was established. TruNatomy orifice modifiers were used to shape the canal orifices and the glide path was achieved with the #17 TruNatomy Gliders. Canal preparation was completed using the #20 TruNatomy Small and #26 TruNatomy Prime shaping files to the working length. The canals were lubricated with 17% EDTA gel and the irrigation protocol was consistent with that used in Group 4.

## Obturation for group 2, 3, 4 and 5:

After complete instrumentation and irrigation, the root canals were dried with sterile absorbent paper points. Following the manufacturer's instructions, AH Plus sealer (Dentsply DeTrey GmbH, Konstanz, Germany) was coated properly on the root canal walls with the help of a lentulospiral. After this the master gutta-percha cone was uniformly coated with the sealer and inserted into the root canal. Using the spreader of suitable size accessory cones was inserted and the root canals were obturated using the lateral condensation technique. Excess gutta-percha was removed and the remaining gutta-percha was condensed properly with the help of a heated plugger 1mm below the canal opening. Orifice of each canal was then sealed with a temporary restorative material (Cavit, Coltene) and the teeth were stored at 37°C in 100% humidity for 2 weeks.

## Simulation of PDL and mounting of samples:

In order to simulate the PDL and create a gap of 0.2mm, 4mm length of external surface of each root was covered with a thin layer of melted wax and dried. The roots were then mounted by embedding them in self-curing acrylic blocks of 25mm x 20mm dimensions. After complete polymerization of the acrylic resin, the roots were removed from the blocks and the wax coating on them was removed using warm water. The simulated acrylic block sockets were filled with light body additional silicone impression material and the roots were immediately reinserted into their respective sockets. The mounted samples were then kept in a damp towel to prevent drying before testing.

## Testing of samples:

All the samples were mounted in Universal testing machine (Asian Test Equipment) and subjected to an axial compression load applied parallel to the long axis of the tooth by means of a conical indenter of 0.5mm diameter and 60° taper running at a crosshead speed of 1mm/min. the load was applied until the samples fractured and the "fracture" in this study is the point at which a sharp drop was observed. This point was verified with the help of a computer attached to the Universal testing machine .The load at which the fracture occurred was expressed in Newtons. The data obtained was recorded, tabulated and statistically analyze. The data collected was subjected to further evaluation by statistical analysis and the results were concluded based on the statistics. Data was presented as mean and standard deviation. Continual variables were compared using Repeated Measure ANOVA test. Significance value was defined by p values less than 0.05 using Two-tailed test. Data analysis was performed using IBM-SPSS version 21.0 (IBM-SPSS Science Inc., Chicago, IL).

## Results:

The mean Fracture Resistance Value (FRV) was highest in the control group, with a value of 473.99 ± 70.62, followed by the Hand files group (397.64 ± 19.02), TruNatomy group (333.48 ± 35.83), Pro-Taper Next group (320.71 ± 45.29) and WaveOne Gold group, which exhibited the lowest value (286.26 ± 12.94). Statistical analysis revealed a significant difference in FRV between the groups (p < 0.0001). Intergroup comparisons indicated that the control group (Group 1) demonstrated the highest fracture resistance (p < 0.05), followed by the Hand files group (Group 2). Groups 3, 4 and 5 (WaveOne Gold, Pro-Taper Next and Trunatomy, respectively) exhibited similar fracture resistance values, with no significant difference among them (p > 0.05); although Group 3 (WaveOne Gold) showed the lowest resistance to fracture (refer to Table 1 and Figure 1 - (see PDF)). The fracture resistance of different file systems was in the following order.

Hand K-Files > TruNatomy > Pro-Taper Next > WaveOne

## Discussion:

The primary objective of endodontic therapy is the complete removal of infected pulp tissue, debris and microorganisms from the root canal system to achieve a hermetic seal of the root canal space [10]. This is accomplished through proper biomechanical preparation and adequate canal enlargement, aided by copious irrigation. Canal shaping occurs through the interaction between the dentinal walls and the endodontic instrument. During these interactions, transient stresses are generated, especially in the middle curved portions of the root canal. These stresses can lead to the formation of dentinal defects such as microcracks and craze lines, which may progress to vertical root fractures. Studies by Oliveira *et al.* [11] and Assif *et al.* [12] demonstrated that endodontically treated teeth are more prone to fractures compared to vital teeth. The weakening of tooth structure is primarily attributed to mechanical stress, instrument kinematics and the geometry of the instruments used during root canal procedures [9]. Kim *et al.* reported a potential association between vertical root fractures and the design of endodontic instruments [9]. Instrument geometry can induce defects in dentin, thereby weakening the tooth and increasing its fracture susceptibility. Stiffer instruments generate greater stresses [13] and factors influencing instrument stiffness include manufacturing techniques, instrument size [13], taper (constant or regressive), cross-sectional design, tip design, helix angle, flute length and pitch variation [14].

This *in vitro* study compared the fracture resistance of endodontically treated teeth prepared with four different file systems: Hand K-Files, WaveOne Gold, Pro-Taper Next and TruNatomy. The study was conducted *in vitro* because the physical changes specifically fracture resistance, induced by various root canal preparation techniques cannot be assessed *in vivo*. Teeth that retain their natural anatomy and mineral content exhibit higher fracture resistance, as they are better equipped to withstand occlusal forces over time compared to teeth subjected to biomechanical preparation [15]. Endodontic treatment renders teeth more brittle and fragile, increasing their susceptibility to fractures under load compared to intact teeth [7]. Krikeli *et al.* (2018) also found that endodontically treated teeth have reduced fracture resistance compared to untreated teeth [16]. Both hand and rotary instruments used for canal preparation remove significant amounts of root dentin due to repeated contact between the instruments and dentinal walls, weakening the tooth and increasing fracture risk [17, 18]. Rotary files, in particular, require more rotations to complete the biomechanical preparation, which may contribute to the formation of microcracks and subsequently increase the likelihood of root fractures [19]. Instrument taper plays a significant role in determining the amount of stress exerted on the root canal walls. A higher taper generates greater stress. Hand files, with a smaller 2% taper, exhibit greater fracture resistance than rotary files, which typically have tapers of 4% or more [20]. The higher taper of the Wave One Gold system (7%) result in increased dentin removal, weakening the tooth and predisposing it to fractures [21]. The reciprocating motion of the WaveOne Gold system, with a 150° clockwise rotation followed by a 30° counter clockwise disengagement, generates torsional forces that contribute to microcrack formation and reduced fracture resistance [21]. The WaveOne Gold system's semi-active cutting tip further increases its aggressiveness in dentin removal compared to the Pro-Taper Next and TruNatomy systems, which feature non-cutting tips [22]. Cassimiro *et al.* (2018) reported that Reciproc, Pro Taper Next and WaveOne Gold produced microcracks in 18.33%, 33.33% and 60% of cases, respectively [23]. The Pro-Taper Next system's off-centered rectangular cross-sectional design, as opposed to the parallelogram cross-section of WaveOne Gold, reduces stress generation [24]. The swaggering motion of the Pro-Taper Next files minimizes taper lock and screw effects, reducing the duration of contact between the file and dentin, which may explain its higher fracture resistance compared to WaveOne Gold [25]. Prior studies by Pawar *et al.* (2014) [26] and Khalap *et al.* (2015) [21] similarly suggested that teeth prepared with Pro-Taper Next exhibit comparable fracture resistance to those prepared with WaveOne. The TruNatomy file system, made from a heat-treated 0.8 mm Ni-Ti wire, improves cutting efficiency and flexibility, thereby preserving radicular dentin. A uniform 4% taper, which is less aggressive than the 6% or greater variable tapers of WaveOne Gold and Pro-Taper Next is shown [27]. This decreased taper conserves more root dentin, contributing to improved tooth strength. The results of this study concluded that Hand K-Files provided the highest fracture resistance among the file systems tested, followed by the TruNatomy system, Pro-Taper Next and WaveOne Gold. These findings are consistent with the studies by Cirakogly *et al.* (2021) [28] and Nassar *et al.* (2022) [29], which found that teeth prepared with the TruNatomy system demonstrated the greatest fracture resistance. The differences in fracture resistance among the systems can be attributed to variations in radicular dentin loss, taper, cross-sectional design, cutting efficiency and rotational speed of the instruments used.

## Limitations:

Despite efforts to minimize discrepancies between experimental settings and clinical conditions, it remains challenging to completely eliminate the influence of external factors on the results. This study was conducted *in vitro* and thus, certain *in vivo* factors, such as masticatory forces and the dynamic oral environment, could not be replicated in the laboratory setting. Additionally, the file systems used in the study operate at specific speed and torque values, which may result in variations in the amount of stress generated and differences in cutting efficiency.

## Conclusion:

Root canal-treated teeth are more prone to fracture due to the loss of radicular dentin, reducing their fracture resistance compared to non-treated teeth. As a result, endodontically treated teeth exhibit lower fracture resistance compared to non-treated teeth. Advances in science and technology have led to the development of various heat-treated rotary file systems designed to enhance fracture resistance during root canal procedures. We show that the highest fracture resistance was observed with Hand K-Files, followed by TruNatomy, Pro-Taper Next and WaveOne Gold. Particularly, the Pro-Taper Next and WaveOne Gold systems demonstrated nearly equivalent fracture resistance.

## Figures and Tables

**Table 1:** Inter group comparison

**S.no**	**FRV**	**Mean**	**Std. Deviation**	**F value**	**P value**
Group 1	Control	473.99	70.62	31.16	<0.0001
Group 2	Hand files	397.64	19.02		
Group 3	WaveOne GOLD	286.26	12.94		
Group 4	Pro-taper NEXT	320.71	45.29		
Group 5	Trunatomy	333.48	35.83		
	Total	362.42	78.29		
